# From molecular decline to regeneration failure: seed aging in European beech (*Fagus sylvatica*) challenges the conservation of forest reproductive material

**DOI:** 10.1093/treephys/tpag121

**Published:** 2026-08-26

**Authors:** Elwira Nawrocka, Hanna Fuchs, Ewelina Ratajczak

**Affiliations:** Department of Developmental Biology, Institute of Dendrology, Polish Academy of Sciences, 62-035 Kórnik, Poland; Department of Developmental Biology, Institute of Dendrology, Polish Academy of Sciences, 62-035 Kórnik, Poland; Department of Developmental Biology, Institute of Dendrology, Polish Academy of Sciences, 62-035 Kórnik, Poland

**Keywords:** desiccation tolerance, DNA methylation, longevity, mitochondrial dysfunction, oxidative stress, silviculture

## Abstract

European beech (*Fagus sylvatica* L.) is a keystone species in temperate forests in Central and Western Europe. However, its regeneration is increasingly constrained by erratic seed production, climate variability and poor storage of its partially desiccation-sensitive seeds. Rapid loss of viability during seed storage poses a major obstacle to the conservation. This review synthesizes current knowledge of the physiological, biochemical and epigenetic processes involved in the aging of beech seeds. Oxidative stress emerges as a central driver: the accumulation of reactive oxygen species (ROS) after seed shedding disrupts membrane integrity, impairs mitochondrial respiration and accelerates programmed cell death, whereas controlled ROS levels also act as essential signaling molecules during germination. Mitochondrial decline progressively reduces energy supply during hydrated storage, while epigenetic instability, particularly DNA hypomethylation, weakens macromolecular repair and germination potential. The decrease in protective molecules, including dehydrins and raffinose family oligosaccharides, together with other aging-related processes, contributes to the rapid decline in beech seed viability during conventional moist storage at low temperatures. We argue that effective conservation strategies must integrate early molecular diagnostics (e.g., ROS accumulation, DNA methylation, mitochondrial respiration) with improved drying, freezing and cryopreservation protocols. By explicitly linking cellular mechanisms of seed aging to regeneration failure and forest-scale resilience, this synthesis underscores the urgent need for adaptive management to safeguard the regeneration potential and ecosystem resilience of *Fagus sylvatica* in a rapidly changing climate.

## Graphical Abstract

**Graphical Abstract:**
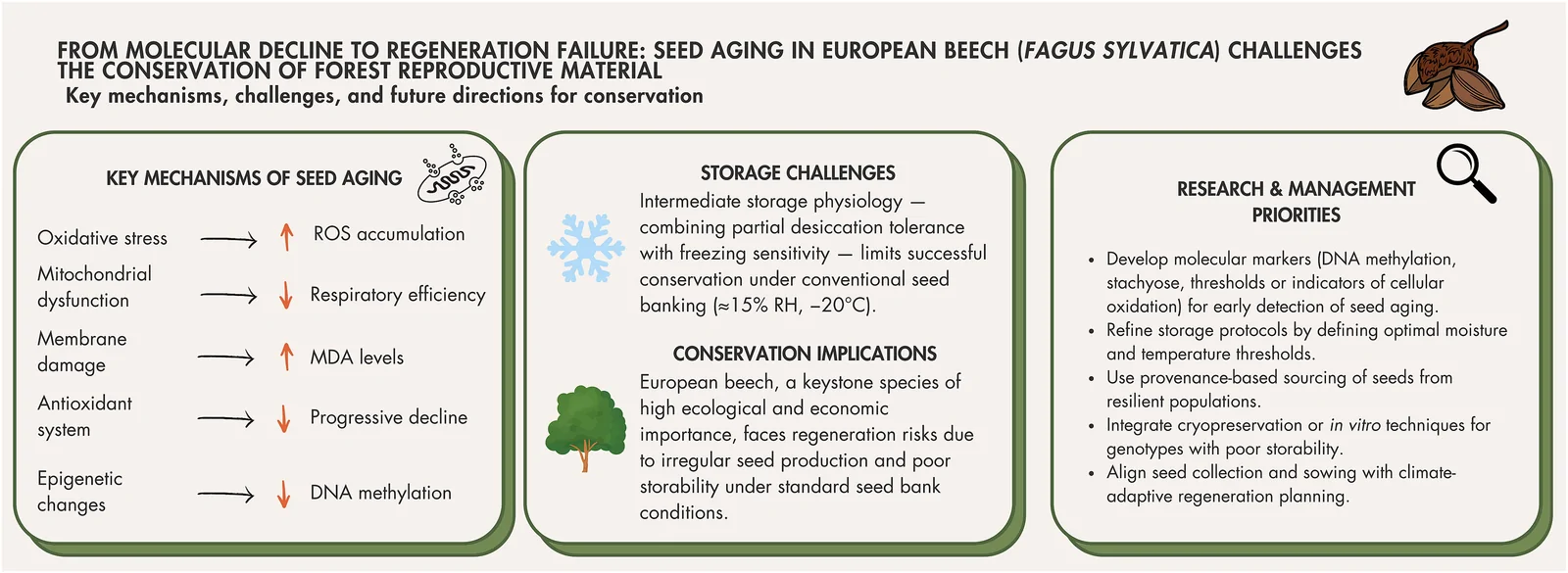

## Introduction

European beech (*Fagus sylvatica* L.) is a dominant broadleaf species across Central and Western Europe, valued both for its ecological role and high-quality timber (Pramreiter and Grabner 2023, Przepióra et al. 2025). Reproduction follows a classic masting strategy, with irregular years of high seed production separated by years of low crops. Mast events are typically synchronized across individuals, enhancing pollination success and overwhelming seed predators through economies of scale. The production of exceptionally large seed can exceed the consumption capacity of seed predators, allowing more seeds to survive and germinate (Kelly et al. 2001, Bogdziewicz et al. 2020*a*). Seed production is strongly shaped by climatic conditions over the two preceding years, with floral induction favored by warm, dry summers, and highest yields often observed after a sequence of a cold, wet summer followed by a warm, dry one (Piovesan and Adams 2001, von Wüehlisch 2008, Gavranović Markić et al. 2024).

Recent climate warming is disrupting these reproductive dynamics. Across Europe, higher summer temperatures are associated with increased average seed production but reduced interannual variability and synchrony in seed production among individuals, a phenomenon described as ‘masting breakdown’ (Bogdziewicz et al. 2020*b*, Foest et al. 2024, 2025). Lower flowering synchrony and subsequent seed production weakens both pollination efficiency and predator satiation, eroding the adaptive advantages of masting (Pearse et al. 2021). At regional scales, climatic heterogeneity has further led to spatial decoupling of mast years, including increasing asynchrony between northern and southern Polish populations (Pawłowski et al. 2024). These shifts in seed production destabilize regeneration dynamics and challenge the continuity of forest reproductive material (FRM) supply, increasing the need for reliable storage of viable beech seeds.

Beyond irregular seed supply, beech seeds present inherent difficulties in long-term preservation. Based on desiccation tolerance (DT) and freezing tolerance (Walters 2015), seeds are classified as orthodox, recalcitrant or intermediate (Roberts 1973, Ellis et al. 1990), and beech seeds belong to the intermediate category (Gosling 1991, León-Lobos and Ellis 2002). Their sensitivity to both desiccation and freezing restricts storage options and increases the risk of viability loss, posing a major challenge for FRM conservation.

Irrespective of storage conditions, prolonged storage leads to seed aging, a cumulative and irreversible process associated with declining viability, delayed germination, and reduced stress tolerance during early seedling growth (Pukacka and Ratajczak 2007, Waterworth et al. 2015, Ebone et al. 2019; Table 1). During imbibition (i.e., initial seed water uptake), seeds activate repair and protective mechanisms that help restore cellular homeostasis (Sano et al. 2016). However, these recovery systems have limited capacity. When damage to critical cellular structures—particularly mitochondria, plasma membranes or genomic DNA—exceeds repair thresholds, mitochondrial dysfunction restricts ATP production, macromolecular repair cannot be sustained, and germination potential is irreversibly lost, even under optimal conditions (Yin et al. 2016, Zhang et al. 2021).

**Table 1:** Cellular and molecular changes during seed aging in European beech.

Level	Change	Potential impact on germination	References
Cellular	Membrane lipid peroxidation and increased permeability	Electrolyte leakage; loss of membrane integrity	Ratajczak et al. 2015*b*, Małecka et al. 2021
Biochemical	Accumulation of lipid peroxidation products (e.g., 4-HNE, MDA) and reactive oxygen species (ROS)	Oxidative stress; activation of cell-death or signaling pathways	Ratajczak et al. 2015*a*, 2015*b*, Schaur et al. 2015, Małecka et al. 2021, Klupczyńska et al. 2022
	Rise in raffinose family oligosaccharides (RFOs)	ROS scavenging and induction of cytoplasmic vitrification; increased tolerance to desiccation and oxidative stress	Ma et al. 2019, Salvi et al. 2022
	Accumulation of heat shock proteins (HSPs)	Stabilization of membranes and proteins; contributing to structural integrity and stress resilience	Kalemba et al. 2019, Salvi et al. 2022
Epigenetic	Altered DNA methylation patterns	Reduced expression of repair and antioxidant genes	Mira et al. 2020, Michalak et al. 2023
	Methylation changes associated with stress-response regulation	Epigenetic activation of defense-related pathways contributing to improved germination and seed vigor	Waterworth et al. 2019, Mira et al. 2020, Michalak et al. 2023

In beech, oxidative stress is a central driver of seed deterioration during storage. Accumulation of reactive oxygen species (ROS) damages membranes, proteins, DNA and RNA (Walters et al. 2026), collectively reducing seed vigor and viability, with the root apical meristem being particularly sensitive (Ratajczak et al. 2015*a*, Kalemba et al. 2019, Małecka et al. 2021). Mitochondria play a key role in seed deterioration, particularly during imbibition following storage, when mitochondrial respiration is reactivated and metabolic activity resumes. Mitochondria dysfunction increases electron leakage and superoxide anion (O₂•^−^) formation. Because hydrogen peroxide (H₂O₂) is more stable than superoxide anion, it can accumulate when detoxification is insufficient, disrupting redox homeostasis and promoting a self-amplifying cycle of oxidative damage (Møller 2001, Noctor et al. 2018). Seed aging during storage is associated with oxidative stress accumulation, which damages cellular components. Storage-induced changes in global DNA methylation correlate with aging and suggest that epigenetic mechanisms modulate seed responses to oxidative stress (Mira et al. 2020, Michalak et al. 2023). Complementary protective mechanisms, including synthesis/activation of molecular chaperones and accumulation of compatible solutes, support cellular homeostasis and influence deterioration rates (Kalemba et al. 2019).

This review synthesizes advances in the physiological, biochemical and molecular mechanisms of seed aging in beech, particularly mitochondrial dysfunction and epigenetic regulation. These processes link directly to the ecological constraints of beech intermediate seed physiology, characterized by limited tolerance to desiccation and freezing, which challenge natural regeneration and long-term conservation under current climate change. By integrating multidisciplinary evidence on the primary drivers of seed deterioration and their interactions, we provide a framework for improved conservation strategies, including ex situ storage optimization and quality monitoring, as well as in situ management to enhance reproduction and recruitment.

## Seed viability as the hidden constraint in beech regeneration

In beech, sexual reproduction culminates in the formation of mature fruits called burs, which develop in autumn and usually contain two, occasionally three, beechnuts. These seeds are enclosed in a four-lobed, woody husk. Once mature, the bur opens while still on the tree, allowing seeds to fall to the ground. Natural seed dispersal is facilitated primarily by gravity (barochory) and animals (zoochory). Many vertebrates consume seeds or store them in the soil, which contributes to secondary dispersal (Chalupa 1996, Seneta and Dolatowski 2025). The seeds are small, brown, trihedral and thinly shelled. They lack endosperm and store nutrients in large, fleshy cotyledons (exalbuminous), which also provide mechanical protection. The embryonic axis is readily visible and, when well developed, consists of the radicle and hypocotyl (Johri et al. 1992, Pukacka and Wójkiewicz 2003).

The water content within the seed is unevenly distributed, with the embryonic axis retaining more moisture than the cotyledons, increasing its vulnerability to freezing injury and oxidative stress. Differential thermal analysis and electron spin resonance studies by Pukacka et al. (2003) confirmed that, compared with cotyledons, the embryonic axis contains freezable water that crystallizes at relatively higher moisture contents and at low temperatures, and requires more extensive dehydration to achieve a glassy state. This asymmetry in DT makes the embryonic axis more prone to aging and loss of viability during storage under dry and cold conditions. Although intracellular glass formation can occur in both cotyledons and embryonic axes, the formation of an intracellular glass alone does not guarantee DT. Desiccation tolerance is associated with several physicochemical properties of the glassy cytoplasm, including reduced molecular mobility, increased cytoplasmic viscosity and the presence of a stable glass matrix. These properties are influenced by tissue water content and macromolecular composition. In orthodox seeds, extensive dehydration promotes the formation of a highly stable, low-mobility glass that immobilizes the cytoplasm and suppresses deleterious biochemical reactions (Buitink and Leprince 2004). In contrast, the higher water content and physicochemical properties of the cytoplasm of recalcitrant seeds hinder the formation of an equally stable glass matrix, limiting its ability to provide comparable protection during drying (Ballesteros et al. 2020). These differences also affect storage behavior. In embryonic axes with relatively high residual water content, reduced glass stability increases the likelihood of water crystallization during dry, low-temperature storage, thereby accelerating aging processes and contributing to loss of viability (Pukacka et al. 2003, Ballesteros et al. 2020).

Seed development follows a typical pattern observed in angiosperms. It begins with fertilization and embryogenesis, followed by maturation. During maturation, the embryo stops growing, storage compounds accumulate, partial DT develops and physiological dormancy is induced (Goldberg et al. 1994, Kalemba et al. 2009). Dormancy is regulated hormonally and requires cold stratification at approximately 3 °C for a minimum of 6 weeks to be broken. Cold stratification facilitates the synchronization of germination timing, a trait that is imperative for the successful establishment of seedlings in temperate forest ecosystems (Suszka et al. 1996, Pukacka and Wójkiewicz 2003, von Wüehlisch 2008, Pukacka and Ratajczak 2014). Cold stratification promotes dormancy release by altering the phytohormonal balance within seeds, notably through a decline in abscisic acid (ABA) and an increase in gibberellins (GA), which jointly modulate seed sensitivity and dormancy depth. In beech, these responses differ between cotyledons and the embryonic axis because the two tissues exhibit distinct physiological and metabolic responses during cold stratification, which may influence the efficiency and outcome of dormancy release (Arc et al. 2011, Staszak et al. 2019, Pawłowski et al. 2024).

The hormonal regulation of the dormancy–germination transition is primarily mediated by the antagonistic action of ABA and GA. Recent molecular studies have shed light on key components of this signaling network. Staszak et al. (2019) demonstrated that beech seeds with deep physiological dormancy exhibit sustained expression of the ABA-responsive transcription factor ABI5 and 14-3-3 regulatory proteins during stratification, with both becoming downregulated as dormancy is released. According to Lopez-Molina et al. (2003), the response to ABA is negatively regulated by ubiquitination through ABI5 binding protein 1, which affects the stability of ABI5. Thus, this latter mechanism could represent a feedback regulatory process of the ABA signaling pathway to fine-tune the level of active ABI5. Similarly, the GA signaling repressor RGL2 shows a decrease in accumulation as seeds transition to germination (Lee et al. 2002, 2010), reflecting its involvement in blocking GA-induced germination-promoting gene expression. Unlike shallow-dormant seeds such as *Arabidopsis*, where these regulators are transiently present, in beech they persist throughout most of the cold stratification period, highlighting differences in hormonal dynamics across dormancy types (Arc et al. 2011, Staszak et al. 2019).

These physiological and molecular findings reinforce the view that dormancy depth in beech seeds is tightly regulated by hormonal balance, with local adaptations modulating the sensitivity and duration of cold stratification. Understanding these mechanisms is crucial for refining both natural regeneration strategies and ex situ conservation protocols in the face of climate change.

In addition to hormonal control, the chemical composition of beechnuts plays a vital role in seed performance. Beech seeds are rich in biologically active compounds, including phenolic compounds, α-tocopherol, sterols, ascorbic acid, glutathione and soluble proteins (Pukacka and Ratajczak 2007, Obranović et al. 2024). Pukacka and Ratajczak (2007) demonstrated that, after storage, germination capacity was strongly correlated with total phenolics, UV-absorbing phenols and soluble proteins. Moderate positive associations were also observed with α-tocopherol and ascorbic acid, whereas sterols and glutathione were not significantly correlated with germinability. These compounds, particularly phenolic and soluble proteins, likely contribute to oxidative stress mitigation, protein protection and membrane stability during storage and early germination, thereby supporting overall seed viability and vigor (Doria et al. 2009, Seal et al. 2010, Adetunji et al. 2021).

### From provenance to plasticity: germination patterns across beech populations

Seed longevity is considered a genetically complex, multigenic trait (Rajjou and Debeaujon 2008, Waterworth et al. 2015, 2024). Intraspecific variation in seed dormancy, quality and seedling vigor is substantial across the range of beech and is closely linked to the maternal environment (Donohue et al. 2010). A study by Pawłowski et al. (2024) of 26 populations in Poland revealed that seeds from northern, warmer and drier sites had deeper dormancy, lower germination rates and produced seedlings with lower biomasses and thicker, shorter roots. Conversely, populations from southern, cooler and wetter environments presented greater seed mass, more rapid and successful germination, and more vigorous seedling growth. While these patterns reflect the influence of the maternal environment on seed traits, complementary evidence from a large reciprocal transplant experiment across Europe showed that germination, establishment, and seedling survival of beech were strongly influenced by local environmental conditions (Muffler et al. 2021). However, no signs of local adaptation were detected, indicating that phenotypic plasticity rather than genetic differentiation plays a dominant role. Collectively, these findings suggest that both maternal effects and phenotypic plasticity interact to shape early regeneration success in beech (Valladares et al. 2014, Benito Garzón et al. 2019).

The key climatic drivers of intraspecific variation in seed traits and early seedling performance include reference evaporation (i.e., atmospheric evaporative demand estimated as reference evapotranspiration), the beginning and end of the frost-free period, and indices of thermal and hydric stress such as the annual and summer heat-to-moisture ratios (Pawłowski et al. 2024). These variables are significantly correlated with germination timing, dormancy depth, and seedling establishment. This climate-related phenotypic differentiation complements earlier findings by von Wüehlisch (2008) of ecotypic variation in seed traits and phenology. Such phenotypic differentiation, driven largely by plasticity rather than fixed local genetic adaptation, is particularly relevant under climate change.

While marginal populations may harbor valuable adaptive traits for future conditions, they often experience environmental stress that limits reproductive output and seed quality, increasing their vulnerability (Hampe and Petit 2005). Therefore, understanding the interaction between seed ecology and climatic adaptation is essential for effective conservation, forest management and assisted migration strategies in a warming world. Recent distribution modeling by Dyderski et al. (2025) confirms that European beech is among the partially threatened tree species in Europe and is projected to lose a significant portion of its current climatic niche by the mid- to late 21st century under severe warming scenarios. However, under moderate climate change scenarios, beech is projected to retain climatic suitability across large parts of its range, particularly in Central and Western Europe. This pattern aligns with variation in dormancy and germination success across temperature and moisture gradients, suggesting that climate-related life-history variation, such as seed mass and wood density, influences the species’ resilience and regeneration potential under future conditions (Hsu et al. 2024, Pawłowski et al. 2024).

### Short- and long-term storage of beech seeds: opportunities and limitations

The optimal conditions for the longevity of beech seeds are typically 7.8–11.5% moisture content (MC) and temperatures between −10 and −20 °C (Gosling 1991, León-Lobos and Ellis 2002, Pukacka and Wójkiewicz 2003). For short-term storage, cold stratification (0–5 °C, ~ 28–32% MC) is commonly applied, allowing prompt seed germination after sowing in spring (Gosling 1991). Long-term storage generally involves moderate drying to ~ 10% MC followed by freezing at −10 °C or lower (León-Lobos and Ellis 2002, Pukacka and Wójkiewicz 2003). Overdrying below 7.8% MC increases the risk of oxidative damage and loss of viability (León-Lobos and Ellis 2002). Germination capacity declines sharply during long-term storage, reaching 66% in 7-year-old seeds and 20% in 10-year-old seeds stored at −10 °C and 7–9% MC. After dormancy release through cold stratification and subsequent drying, beech seeds cannot be stored as long as seeds of other non-dormant species (Pukacka and Ratajczak 2007).

Cryopreservation—storage at liquid nitrogen temperatures (~ −196 °C)—offers a promising alternative, for long-term conservation of seeds with intermediate physiology. Cryopreservation protocols aim to achieve ice-free vitrification of the cytoplasm, resulting in the formation of a glassy state that prevents intracellular ice formation. Protocols involving vitrification and freeze-drying have been tested for embryonic axes, though success varies due to the sensitivity of embryonic tissues to dehydration and freezing (Li and Pritchard 2009, Walters et al. 2013, Pammenter and Berjak 2014, Pukacka and Ratajczak 2014). As noted by Ballesteros et al. (2020), intermediate seeds such as beech seeds require carefully adjusted desiccation and cooling rates, often with the addition of cryoprotectants, to minimize physiological damage. These refined protocols are employed in advanced seed banks, such as the Millennium Seed Bank, for species that do not survive conventional −20 °C storage (Hay and Probert 2013, Walters et al. 2013, Ballesteros et al. 2021).

In parallel, in vitro micropropagation methods are emerging as complementary tools in ex situ conservation. Zahn et al. (2025) developed shoot-tip and nodal explant culture systems for beech which presented genotype-dependent growth and survival responses. Modified nutrient media and light regimes increased culture longevity, suggesting the potential for ex situ conservation of genetically valuable material that cannot be preserved via conventional seed storage alone. Hazubska-Przybył et al. (2015) also demonstrated successful in vitro survival of beech explants, particularly embryonic axes and zygotic embryos, juvenile explants with undifferentiated or partially differentiated tissues and high regenerative capacity under in vitro conditions, highlighting the potential of this species for clonal propagation under controlled conditions.

### Post-storage decline in seed performance: a challenge for FRM conservation

Beech seed membranes contain polyunsaturated fatty acids (PUFAs), which are particularly susceptible to oxidative attack because of their multiple double bonds (Bailly 2004, Ayala et al. 2014, Pukacka and Ratajczak 2014). Lipid peroxidation generates reactive electrophile species (RES), such as malondialdehyde (MDA) and 4-hydroxy-2-nonenal (4-HNE), which further amplify oxidative damage and propagate lipid peroxidation chain reactions (Ayala et al. 2014, Gerna et al. 2022, Cai et al. 2025). In this context, RES result from storage-related oxidative processes and exhibit a dual role in seed physiology, acting both as cytotoxic agents and as signaling molecules involved in the regulation of gene expression and the disruption of cellular homeostasis.

Redox disruption is increasingly recognized as a key interface between metabolic stress and epigenetic regulation. The activity of DNA methyltransferases and other chromatin-modifying enzymes depends on cellular redox status, as oxidative conditions can alter the availability of key cofactors and modify redox-sensitive thiol groups, thereby affecting enzymatic activity and making the epigenome responsive to redox fluctuations (Bräutigam et al. 2013, Zhang et al. 2018). Consistently, Michalak et al. (2023) demonstrated that storage-associated deterioration in lipid-rich beech seeds is accompanied by changes in global DNA methylation, indicating that aging-related redox shifts may translate into altered epigenetic states.

For FRM conservation, this perspective shifts the focus from storage conditions alone to the molecular consequences of lipid-rich seed, particularly the susceptibility to lipid peroxidation and the associated formation of RES. Integrating conventional storage optimization with biochemical and epigenetic monitoring of seed redox status, lipid peroxidation products and DNA methylation patterns may help identify markers of deterioration and resilience, ultimately supporting strategies aimed at maintaining the reproductive capacity and adaptive potential of *F. sylvatica* under increasing environmental variability.

### Oxidative stress as a central driver of seed aging and loss of viability

The process of aging in beech seeds is closely associated with the accumulation of ROS, particularly O₂•^−^, H₂O₂ and hydroxyl radicals (•OH). Reactive oxygen species affect membranes, proteins and nucleic acids, and can induce oxidative stress and programmed cell death (PCD), ultimately reducing germination potential (Ratajczak et al. 2015*b*, Małecka et al. 2021). The overproduction of ROS during storage is especially evident in embryonic axes, where metabolic reactivation during imbibition begins, making these tissues particularly vulnerable. Ratajczak et al. (2015*b*) provided detailed quantitative and spatial analyses of ROS accumulation in beech seeds stored under controlled −10 °C for up to 13 years. They reported that H₂O₂ and O₂•^−^ levels increased nearly two- and threefold, respectively, in embryonic axes over time. The accumulation of ROS was negatively correlated with electrolyte leakage, indicating compromised membrane integrity. Moreover, •OH production also increased significantly with storage time and during imbibition.

Fluorescence imaging revealed that ROS initially localized in the root cap but progressively spread to the quiescent center and root apical meristem in older seeds—tissues critical for root growth and seedling establishment (Kalemba et al. 2019). These changes coincided with a pronounced decline in catalase (CAT) activity after 5 years of storage, reducing the capacity of the seeds to detoxify H₂O₂ (Ratajczak et al. 2015*b*, Małecka et al. 2021).

DNA fragmentation has also been detected in embryonic axes stored for five years or longer, consistent with ROS-induced genetic damage (Chen et al. 2012, Yin et al. 2016, Pagano et al. 2017). However, these studies did not explicitly report the occurrence of internucleosomal DNA laddering, a hallmark of PCD-like processes, suggesting that the observed DNA degradation may reflect predominantly oxidative stress damage rather than a PCD-like programme. In addition to strand breaks, oxidative stress induces base modifications such as 8-oxo-7,8-dihydroguanine (8-oxoG), as well as DNA adducts formed with lipid peroxidation products (e.g., MDA), which may interfere with replication and repair (Marnett 1999, Chen et al. 2012). Strong correlations between ROS accumulation, CAT activity and DNA degradation further confirm oxidative stress as a key factor driving loss of seed viability during long-term storage (Ratajczak et al. 2015*a*, Klupczyńska et al. 2022).

To counteract oxidative damage, beech seeds deploy antioxidant defense including non-enzymatic low-molecular-weight antioxidants (ascorbate, glutathione) and enzymatic scavenging systems such as CAT and peroxidases (Kalemba et al. 2021, Małecka et al. 2021). Notably, when seed MC drops below ~ 10% and storage occurs at subzero temperatures (e.g., −20 °C), cells are likely to enter a glassy state (Pukacka et al. 2003). In this state, molecular mobility, enzymatic activity, and long-distance diffusion are severely restricted. Although ROS generation may still occur, the efficiency of enzymatic detoxification is markedly reduced, and protection against oxidative damage relies predominantly on non-enzymatic antioxidants and the physicochemical stabilization of cellular structures (Gerna et al. 2022).

However, as shown in multiple studies (Pukacka and Ratajczak 2005, 2007, 2014, Ratajczak et al. 2019*b*), antioxidant protection decreases with storage time, including under controlled conditions that maintain seeds in a glassy state. For example, although the total glutathione pool remains relatively stable, a decrease in the protein-bound thiol fraction has been observed, reflecting progressive thiol oxidation associated with the conversion of reduced glutathione (GSH) to its oxidized form (GSSG) and altered protein glutathionylation (Pukacka and Ratajczak 2014). Consistently, the ratios of redox couples such as GSH/GSSG and ascorbate and dehydroascorbate (ASA/DHA), shift toward more oxidized states during prolonged storage and subsequent imbibition, especially in non-dormant seeds and embryonic axes. In beech seeds, ROS levels were assessed before storage and after defined storage conditions (4 and 20 °C; 45 and 75% RH), revealing progressive oxidative changes during the storage period (Pukacka and Ratajczak 2014).

Peroxiredoxins (PRXs), a class of thiol-based peroxidases, are important components of redox regulatory networks in seeds and contribute to the maintenance of redox homeostasis during aging and stress (Klupczyńska et al. 2022). Among the six identified PRX isoforms in beech seeds, 2CysPRX and mitochondrial PRXIIF were most abundant during seed development and remained detectable after long-term storage, suggesting a role in maintaining redox homeostasis under these conditions (Ratajczak et al. 2019*b*). In contrast, 1CysPRX, a peroxiredoxin involved in the reduction of lipid hydroperoxides and regulation of cellular redox balance, appeared largely inactive in stored seeds, potentially limiting the detoxification capacity and contributing to desiccation sensitivity (Illing et al. 2005, Ratajczak et al. 2019*b*).

Additional evidence from Pukacka and Ratajczak (2014), based on dormant and non-dormant beech seeds stored under controlled temperature (4 and 20 °C) and relative humidity (45 and 75% RH) conditions highlighted that non-dormant seeds, although initially viable, are more susceptible to oxidative damage during storage. This is particularly evident at elevated temperature (20 °C), where increased ROS production coincides with higher electrolyte leakage and enhanced accumulation of free fatty acids, reflecting progressive membrane deterioration. These changes are accompanied by a decline in membrane phospholipids (phosphatidylcholine [PC], phosphatidylethanolamine [PE]), increased levels of GSSG, and reduced α-tocopherol and phenolic antioxidants, underscoring the extent of oxidative damage to cellular membranes, particularly in embryonic axes.

Focusing on seed dormancy, Pawłowski (2007) investigated beech seeds during dormancy-breaking and early germination-related transitions, comparing treatments with ABA and gibberellic acid (GA₃). In this context, superoxide dismutase and ascorbate peroxidase were identified as hormone-responsive proteins, suggesting that redox regulation is integrated with hormonal signaling during early developmental transitions.

Collectively, current evidences in beech indicate that seed aging is associated with a progressive decline in antioxidant protection and redox buffering capacity, particularly in embryonic axes. Reactive oxygen species accumulate in meristematic tissues, while cellular redox homeostasis gradually shifts toward a more oxidized state, contributing to the loss of viability (Pukacka and Ratajczak 2014, Ratajczak et al. 2015*b*, Małecka et al. 2021, Klupczyńska et al. 2022). Monitoring ROS levels, PRX expression, the GSH/GSSG ratio and other redox-related changes may therefore provide useful markers of seed storability and support the optimization of conservation strategies.

### Molecular protection and its role in desiccation tolerance

To withstand drying and post-dispersal survival, beech seeds synthesize a suite of protective molecules during maturation. Among these, late embryogenesis abundant (LEA) proteins, particularly the hydrophilic subgroup known as dehydrins, play key roles in DT (Szlachtowska and Rurek 2023). In beech, several studies have documented dehydrin accumulation in the embryonic axis during late seed development. Kalemba and Litkowiec (2015) identified two major dehydrin isoforms (26 and 44 kDa) that accumulate in the embryonic axes of developing beech seeds. Both proteins were intrinsically disordered and phosphorylated, suggesting functional plasticity via interactions with membranes, proteins and nucleic acids. Their accumulation increased after the 16th week after flowering, in parallel with tissue dehydration, indicating a potential role in stabilizing macromolecules during desiccation.

Further insights into subcellular dynamics were provided by Kalemba et al. (2015), who examined the localization of dehydrin proteins in various cellular compartments. During seed maturation, dehydrins are found along the plasma membrane, within cytoplasmic vesicles, inside the nucleus (associated with dispersed chromatin), and on the surfaces of mitochondria and amyloplasts. In contrast, their distribution in 2-year-old dry seeds that had been stored at −10 °C in closed plastic boxes at 8–9% water content was more limited, with localization primarily observed in amyloplasts and occasionally in nuclei. Given that dehydrins stabilize membranes, cytoplasmic proteins, and nucleic acids under dehydration and oxidative stress, their restricted subcellular distribution may limit their overall protective capacity. This suggests a reduced but persistent protective role of dehydrins during dry storage (Kalemba et al. 2019, Szlachtowska and Rurek 2023).

Kalemba and Pukacka (2008) extended these findings by assessing changes in LEA and LEA-like proteins during long-term seed storage (1–8 years). They confirmed that the 26- and 44-kDa dehydrin isoforms persisted during storage, and that the abundance of the 26-kDa protein, in particular, was strongly correlated with seed germination capacity. Additional LEA-like proteins (35 and 40 kDa) were detected exclusively in embryonic axes, further underscoring tissue-specific protective mechanisms. A general decline in total soluble protein content, especially in LEA-type proteins, was observed in aged seeds, likely reflecting oxidative degradation or reduced protein stability. LEA-type proteins, particularly in embryonic axes, are thought to maintain cellular integrity by preventing protein aggregation, stabilizing membranes and possibly influencing chromatin architecture. Bioinformatic analyses of beech dehydrins indicate that some sequences contain positively charged motifs, including an RKKK track, which are predicted to interact with DNA and suggest potential nuclear roles during dehydration (Kalemba et al. 2015).

While LEA proteins contribute directly to stress tolerance, Kalemba and Pukacka (2008) also reported the accumulation of a 22 kDa small HSP (sHSP) in aged seeds. This sHSP increased with storage time but was negatively correlated with germination, suggesting stress-induced accumulation rather than a protective effect per se. Nevertheless, sHSPs may still assist in chaperoning denatured proteins or mitigating oxidative stress, particularly in embryonic axes. Notably, mitochondrial HSPs can modulate ROS production in a temperature-dependent manner, thereby influencing seed germination (Ma et al. 2019).

In summary, the dynamic changes in LEA and dehydrin proteins during beech seed development and storage reflect a complex protective network. These proteins act as molecular shields, stabilizers and possible regulators, safeguarding cellular structures under water deficit and oxidative stress conditions. The abundance and localization patterns of these proteins could serve as valuable markers of seed quality and aging status in ex situ conservation programs.

The accumulation and maintenance of non-reducing sugars, particularly raffinose family oligosaccharides (RFO), contribute to DT and viability in intermediate seeds such as beech, as well as in orthodox seeds (Salvi et al. 2022). Although these sugars play an important role in the formation of the glassy state, they are not sufficient on their own to ensure protection against desiccation damage. Increasing evidence indicates that the stability of the intercellular glass depends on complex interactions between sugars and other cellular constituents, including proteins, organic acids, and ions, which collectively determine its physicochemical properties (Buitink and Leprince 2004, 2008). Thus, DT is a multifactorial trait rather than being governed by a single component.

During late maturation of seeds, levels of RFO such as stachyose increase, coinciding with the onset of DT. Over long-term storage, stachyose gradually decreases, while sucrose increases, reflecting a shift in the sucrose to RFO mass ratio which correlates with reduced seed viability. The activity of α-galactosidase, responsible for RFO degradation, also rises during storage, potentially compromising cytoplasmic protection. Together, these findings highlight the role of RFO in contributing to DT and long-term seed viability in beech (Pukacka et al. 2009), through interactions with cellular structures such as membranes and proteins.

The integrity of cellular membranes is a primary determinant of seed viability during storage. In beech seeds, the loss of germinability is closely associated with increased membrane permeability and the progressive degradation of lipid components. As demonstrated by Ratajczak and Pukacka (2005), storage under conditions of elevated temperature and humidity accelerated the loss of viability. This process was accompanied by elevated levels of electrolyte leakage and the accumulation of lipid hydroperoxides (LHPO), which are markers of oxidative damage to unsaturated fatty acids. Lipid peroxidation was strongly and negatively correlated with germination capacity. Similar relationships between membrane deterioration, oxidative damage and seed viability have been widely reported across both orthodox and intermediate seeds (Walters 1998, Corbineau 2024).

Phospholipid analysis by Ratajczak and Pukacka (2005) revealed significant decreases in the levels of PC, PE, phosphatidylinositol (PI) and other key membrane lipids, particularly in embryonic axes, during long-term storage. PUFAs, especially linoleic (18:2) and linolenic acids (18:3), were most affected, consistent with their high susceptibility to lipid peroxidation, reducing the degree of unsaturation and likely impairing membrane fluidity. Despite lipid degradation, no accumulation of free fatty acids was detected, suggesting rapid downstream turnover. At the same time, the levels of α-tocopherol, a lipophilic antioxidant involved in protection against lipid peroxidation (Waterworth et al. 2015, Gerna et al. 2022), and membrane-stabilizing sterols decreased significantly, likely increasing membrane susceptibility to oxidative damage.

Additional insights from Pukacka and Ratajczak (2007) confirmed the central role of membrane-associated antioxidants in seed protection. Over 10 years of storage, declines in total phenolics, UV-absorbing phenols, and α-tocopherol were correlated with reduced seed viability, particularly in embryonic axes, reflecting their role in limiting ROS-induced membrane damage.

Conversely, the sterol and glutathione contents did not correlate with germination capacity. The decrease in ascorbic acid was moderate, with only a modest correlation with viability. These findings were further supported by Pukacka and Wójkiewicz (2003), who examined the impact of drying temperature on beech seed storability. Seeds dried at 30 °C, despite reaching the same final MC as those dried at 15 °C, presented a lower germination capacity, greater electrolyte leakage and increased LHPO levels. A marked reduction in PC and PE, as well as in PUFAs (18:2, 18:3), was observed, particularly in the embryonic axes. These structural changes coincided with a significant decrease in membrane-bound α-tocopherol and sterols, weakening antioxidant defenses. Notably, while free fatty acids were not detected, the accumulation of LHPO, representing primary lipid peroxidation products, indicated enhanced oxidative degradation of membrane lipids. In parallel, the presence of secondary products such as MDA, together with shifts in ascorbate and glutathione redox status, confirmed the occurrence of oxidative stress promoted by elevated temperatures during the drying process.

These results confirm that not only storage conditions but also pre-storage drying protocols critically influence membrane integrity and seed longevity. Overall, molecular protection, including antioxidant systems, non-reducing sugars and dehydrins, plays a central role in conferring DT in beech seeds. From a practical perspective, this suggests that improving FRM quality requires careful optimization of drying temperature and humidity, together with the use of physiological markers, such as membrane integrity, lipid peroxidation and redox balance, to assess seed physiological state and guide storage and reforestation strategies.

### Mitochondrial impairment in seed aging and loss of viability

Mitochondria are central to energy metabolism and redox regulation, and they represent major sites of ROS production. During seed aging, mitochondrial structure and function gradually deteriorate, increasing oxidative stress and contributing to loss of viability (Kurek et al. 2019, Ratajczak et al. 2019*a*). Reactive oxygen species-mediated damage to mitochondrial membranes disrupts oxidative phosphorylation, generating additional ROS and establishing a destructive feedback loop (Małecka et al. 2021). In beech seeds, prolonged storage is associated with compromised mitochondrial ultrastructure, elevated oxidative damage, and reduced germination capacity (Ratajczak et al. 2019*a*, Małecka et al. 2021).

Subcellular analyses of mitochondria isolated from cotyledons and embryonic axes of beech seeds stored under controlled dry conditions (7–8% water content, −10 °C, sealed containers) for 8, 11 and 17 years showed a storage-dependent increase in H₂O₂ and MDA, consistent with progressive oxidative modifications of mitochondrial components reported in aged seeds (Małecka et al. 2021). Seeds were analysed in a dry state without prior germination or imbibition treatment, and mitochondrial fractions were isolated directly from stored embryonic tissues. Confocal imaging demonstrated progressive alterations in mitochondrial morphology, including reduced cristae density and structural disorganization, consistent with age-dependent mitochondrial deterioration. These changes coincided with a marked decline in CAT activity, suggesting a decline in antioxidant capacity associated with mitochondria. Lipid peroxidation products, notably MDA and 4-HNE are widely used markers of lipid peroxidation and oxidative stress, although they are not specific to mitochondria (Ayala et al. 2014). Malondialdehyde can form covalent adducts with DNA bases, particularly guanine and adenine, generating mutagenic lesions such as pyrimidopurinone adducts (e.g., M1G) that can mispair during replication and lead to point mutations (Marnett 1999). In contrast, 4-HNE preferentially modifies proteins by covalently binding to cysteine (Cys), histidine and lysine residues, forming 1,4-Michael type adducts with soft nucleophiles (Schaur et al. 2015). Together, these compounds reflect extensive oxidative damage associated with impaired mitochondrial function and reduced seed viability.

Functional consequences of these structural changes have been demonstrated using high-throughput mitochondrial respiration measurements. Mitochondria isolated from embryonic axes of seeds stored for 15 years showed consistent declines in phosphorylating and non-phosphorylating oxidation and maximal respiration rates, with severe mitochondrial dysfunction including uncoupling and reduced respiratory control ratio (Fuchs et al. 2023). In addition to ROS, mitochondrial dysfunction is closely linked to accumulation of reactive electrophile species (RES), particularly lipid peroxidation-derived α,β-unsaturated aldehydes such as 4-HNE. At higher concentrations, 4-HNE can compromise ATP production, promote mitochondrial permeability transition and trigger programmed cell death pathways; at lower concentrations, it may act as a redox signaling molecule, inducing antioxidant and stress-responsive pathways (Ayala et al. 2014, Biswas et al. 2020, Sharma et al. 2022). Experimental evidence from tobacco BY-2 cells illustrates this dual role: H₂O₂ treatment elevates 4-HNE levels by inhibiting NADPH-dependent carbonyl reductases, leading to cytotoxic accumulation and activation of proteolytic cascades (Biswas et al. 2020).

Many mitochondrial alterations observed in aged seeds appear predominantly under suboptimal storage conditions involving elevated temperature or moisture, which allow residual metabolic activity and oxidative stress (Ratajczak et al. 2019*a*, Małecka et al. 2021). Under conventional seed banking conditions, seeds are maintained in a glassy state characterized by near-complete metabolic arrest, and mitochondrial respiration is largely undetectable (Buitink and Leprince 2004, Rajjou et al. 2012, Walters 2015). Consequently, several age-related mitochondrial changes are likely to manifest primarily during rehydration rather than during quiescent dry storage (Arc et al. 2011, Ratajczak et al. 2019*a*). Accordingly, although Seahorse-based respiration assays provide direct insights into mitochondrial function under controlled laboratory conditions, their applicability to conventional long-term seed banking remains limited (Ayala et al. 2014, Fuchs et al. 2023).

Although mitochondrial impairment is typically discussed in the context of long-term storage, mitochondrial function and oxidative status may also serve as integrative indicators in FRM systems. In particular, these parameters may inform seed maturation dynamics, optimal harvest timing, and comparative assessment of seed lot quality across individual trees and stands. Such applications are consistent with current approaches in forest seed science, which emphasize the importance of physiological markers for evaluating seed quality and storability under operational conditions (Cai et al. 2025).

### Epigenetic regulation of seed aging: insights from DNA methylation

DNA methylation is an enzymatic, post-replicative modification that imposes stable, heritable changes on gene expression without altering the underlying DNA sequence. In higher eukaryotes, it most commonly involves the addition of a methyl group (–CH₃) to the carbon at position 5 of the cytosine ring, forming 5-methylcytosine (5-mC; Stein et al. 2024). DNA methylation typically silences gene transcription, contributes to chromatin organization, and safeguard genome integrity. DNA methylation is increasingly recognized as a key epigenetic mechanism by which plants modulate developmental programs and respond to environmental stress (Gallego-Bartolomé 2020, Lucibelli et al. 2022). In beech, changes in cytosine methylation have been linked to seed desiccation sensitivity, aging and post-storage viability, making cytosine methylation a promising molecular marker for assessing seed physiological state.

Michalak et al. (2023) demonstrated that global DNA methylation, measured as 5-mC level, decreases progressively during desiccation and aging, particularly in embryonic axes. Fresh seeds presented relatively high 5-mC levels (~13.8% in embryonic axes and ~ 14.3% in cotyledons). Desiccation to 10.5% MC resulted in a significant decrease in methylation in embryonic axes, whereas cotyledons remained more stable. Importantly, 5-mC levels in embryonic axes, but not in cotyledons, correlated positively with seed viability. These findings demonstrate that changes in 5-mC levels in the embryonic axis constitute a sensitive epigenetic marker of seed viability in response to desiccation.

Storage conditions also influence global methylation levels. Seeds stored for 3 years at higher moisture content (13.4% MC) and suboptimal temperatures (3 °C or − 3 °C) showed pronounced reductions in both global 5-mC level and germination capacity. In contrast, seeds stored at 7.6% MC and −10 °C maintained stable methylation and viability. Under these conditions, seed performance was similar to orthodox seeds (Michalak et al. 2023). The parallel decline in methylation and viability under suboptimal storage indicates that epigenetic alterations track physiological deterioration and may precede visible losses in germination. Additionally, epigenetic differentiation among beech provenances has been shown to be comparable to genetic variation, suggesting that DNA methylation may contribute to long-term environmental adaptation and inform the selection of seed sources in FRM management (Guevara et al. 2022).

Additional insights into the plasticity of DNA methylation in beech were provided by Nuc et al. (2016), who examined changes in embryonic axes following cryopreservation. Dehydration and vitrification, followed by immersion in liquid nitrogen, caused a transient increase in global DNA methylation (44–48% after 30 days of in vitro culture). Methylation levels subsequently declined to ~ 13.7% after 120 days, matching the baseline levels observed in untreated control axes. These dynamics suggest that methylation acts as a reversible regulatory mechanism in response to extreme abiotic stress. Notably, changes were more pronounced in beech than in oak, reflecting species-specific differences in epigenetic resilience and recovery potential (Nuc et al. 2016). In addition to species-specific studies, recent reviews have emphasized the broader regulatory role of epigenetic mechanisms, including DNA methylation, histone modifications and chromatin remodeling, in somatic embryogenesis and regeneration capacity within recalcitrant plant species (Klupczyńska and Ratajczak 2021, Hesami et al. 2024).

These mechanisms not only govern the expression of key developmental regulators (e.g., LEC1, BBM, WUS; Nic-Can et al. 2013, Karim et al. 2018) but also influence transformation and propagation success. Understanding the mechanisms of epigenetic regulation may offer new strategies to overcome regeneration bottlenecks in intermediate- and recalcitrant-seeded species such as beech. Collectively, these studies highlight that DNA methylation in beech, especially within embryonic axes, represents a dynamic and sensitive marker of DT, aging and stress adaptation (Muffler et al. 2021). Its responsiveness to storage conditions, tissue specificity and correlation with viability underscore its relevance for monitoring seed health and improving long-term conservation strategies for intermediate seed types.

## Conclusions

Seed aging in European beech results from a progressive loss of redox homeostasis and molecular stability rather than from a single dominant failure point. Oxidative processes, mitochondrial dysfunction, depletion of protective macromolecules and epigenetic alterations interact across organizational levels, linking molecular deterioration with whole-seed performance. This integrative view helps explain why intermediate seeds, which only partially tolerate desiccation, show limited longevity compared with orthodox species.

A key conceptual outcome of this review is that many molecular changes described as ‘damage’ may initially represent regulatory or stress-response processes that, beyond certain thresholds, shift toward irreversible deterioration. This highlights seed aging as a dynamic continuum rather than a strictly degenerative process. Understanding where this transition occurs remains a major knowledge gap.

From an applied perspective, the convergence of physiological and molecular indicators suggests that early markers of viability loss may be informative. Redox-related homeostasis, mitochondrial performance after rehydration and epigenetic signatures in embryonic tissues appear particularly promising, yet their predictive value under operational storage conditions still requires validation. Bridging laboratory-based mechanistic studies with real seed banking practice is therefore a priority.

Future research should focus on defining critical thresholds in redox imbalance, clarifying how glassy-state constraints modify aging trajectories and determining whether molecular markers can be predicted. Such integration of mechanistic and applied perspectives is essential for improving conservation strategies for intermediate seeds and for safeguarding the regenerative capacity of beech forests under changing climatic conditions.

## Data Availability

No data were used for the research described in the article.
